# Molecular characterization of methicillin-resistant *Staphylococcus aureus *isolated from tertiary care hospitals 

**DOI:** 10.12669/pjms.304.4946

**Published:** 2014

**Authors:** Atif H. Asghar

**Affiliations:** 1 Atif H. Asghar, Department of Environmental and Health Research, The Custodian of The Two Holy Mosques Institute of Hajj and Umrah Research, Umm Al-Qura University, P.O. Box: 6287, Makkah, Saudi Arabia.

**Keywords:** *MRSA*, *Staphylococcus aureus*, *SCCmec*, *PVL*, *mecA*

## Abstract

***Background and*** ***Objectives:*** Methicillin-resistant *S.* *aureus *(MRSA) tends to be resistant to multiple antibiotics. Methicillin resistance is conferred by the acquisition of the *mec*A gene, which is carried by a mobile genetic element called the staphylococcal cassette chromosome *mec* (SCC*mec*). There are five major types of SCC*mec* elements (I–V). The majority of hospital-acquired MRSA (HA-MRSA) strains carry SCC*mec* types I, II, or III, whereas community-acquired MRSA (CA-MRSA) strains carry SCC*mec* types IV or V. In addition, Panton-Valentine Leucocidin (PVL) is a gene encoding a powerful cytotoxin that is strongly associated with CA-MRSA strains. The present study was aimed to identify the types of SCC*mec* and PVL genes among clinical MRSA isolates.

***Methods:*** This study was conducted in 5 tertiary care hospitals in Makkah city from March to September of 2012. A total of 206 *S. aureus* clinical isolates were analysed using standard microbiological methods. Multiplex PCR was performed on genomic DNA from MRSA isolates in order to identify the types of SCC*mec*. In addition, PCR was performed to detect the PVL gene among the isolates.

***Results:*** Of the 206 *S. aureus* isolates, 114 (55.3%) were MRSA, and 100 of the MRSA isolates carried the *mec*A gene. Results from SCC*mec *typing revealed that 3% were type I; 9% were type II; 47% were type III, and 29% were type IV. Nineteen per cent of the isolates harboured the PVL gene. Furthermore, there was a statistically significant correlation between the presence of the PVL gene and SCC*mec* type IV.

***Conclusion:*** The virulence of MRSA strains is increasing in both hospital and community settings in Makkah, highlighting the importance of their rapid identification in order to appropriately control infection.

## INTRODUCTION

*Staphylococcus aureus* (*S. aureus*) is an important human pathogen that is transmitted in both hospitals and the community. MRSA is a major challenge to hospitals all over the world due to the emergence and spread of isolates with decreased susceptibilities to several antibiotics classes including methicillin and other members of ß-lactam family. MRSA is often sub-categorized as HA-MRSA or CA-MRSA. MRSA is developed by multiple insertions of SCCmec into successful methicillin-susceptible *S. aureus* (MSSA) lineages. The resistance of the organism is due to the acquisition of the methicillin resistance gene *mec*A, coding for the low-affinity penicillin-binding protein (PBP2A). Previously, five major types of SCC*mec* elements (I–V) and several variants there have been identified based on the *mec* gene complex and *ccr* gene allotypes. CA-MRSA strains carry SCCmec type IV or V, whereas the majority of HA-MRSA strains carry SCCmec type I, II or III.^1^^,^^2^

Various molecular typing techniques have been developed for investigating the spread and evolution of MRSA, the most common of which is SCC*mec *typing. Oliveira *et al*. developed a multiplex PCR method for the detection of SCC*mec *types I through IV using *mec*A and different loci on SCC*mec*.^3^ Zhang *et al*. developed a multiplex PCR method for the characterization of SCC*mec *types I through V.^4^ MRSA has spread globally since it was first described in the early 1960s and is currently a major cause of nosocomial infections worldwide.^5^

Some *S. aureus *strains produce Panton-Valentine Leucocidin (PVL), a powerful cytotoxin in human and rabbit mononuclear cells. It has been reported that the PVL gene is strongly associated with CA-MRSA strains worldwide. CA-MRSA infections have mostly been associated with staphylococcal strains bearing the SCC*mec* type IV element and PVL genes.^6^ Studies showed that the presence of PVL in *S. aureus* associated with increased disease severity, ranging from cutaneous infection requiring surgical drainage to severe chronic osteomyelitis, and deadly necrotizing pneumonia.^7^ The present study aimed to identify the types of *SCCmec* and the PVL gene among clinical MRSA isolates collected from Makkah hospitals.

## METHODS

This study was conducted in the five major tertiary-care hospitals from March to September 2012. A total of 206 non-duplicated clinical isolates of *S. aureus* were identified in these hospitals during the study period using standard microbiological methods. These methods included colony morphology on blood and mannitol agar, Gram stain, and catalase and coagulase tests. The collected strains were stored at -86°C in brain-heart infusion containing 15% glycerol until use. Detection of MRSA was carried out using the oxacillin agar screen test according to Clinical Laboratory Standards Institute (CLSI) guidelines. The presence of any growth (>1 colony) was defined as oxacillin or methicillin resistant.^8^

For DNA extraction, a single colony was taken from a nutrient agar plate (Oxoid) after overnight incubation. Cell suspensions were centrifuged at 4,500 rpm for 5 minutes at 4°C. Cell pellets were washed with 1 mL of TE (10 mM Tris pH 8, 10 mM EDTA) and resuspended in 100 µL of TE. After the addition of 50 µL of 10% sodium dodecyl sulphate (SDS), the mixture was incubated for 30 min at 65°C. The lysates were centrifuged, and supernatants were removed. The microtubes were then placed in a microwave oven and heated three times for 1 min each at 750 W. The pellets were dissolved in 200 µL of TE and were extracted with an equal volume of phenol/chloroform/isoamyl alcohol (25:24:1) for 15 min. The aqueous phase was recovered by centrifugation for 20 min, precipitated with ethanol, and resuspended in 50 µL TE**.**^9^ Mutiplex PCR was performed for the detection of the SCC*mec* gene. Table-I lists the primers used to amplify the SCC*mec* gene. Two sets of primers were used. The first set (Oliviera primers) was designed for the typing of SCC*mec* types I–IV based on selected loci (A through F) upstream and downstream the *mecA* gene.^3^ The second set (Zhang primers) was designed for the typing of SCC*mec* types II, III, and V.^4^ For detection of the PVL gene, a single PCR was performed. Table-I lists the primers used to amplify the PVL gene. The 50-µL PCR mixture contained 8 µL of DNA template, 1 µL (100 pmol) of each primer, and a 25 µL of Taq PCR Master Mix polymerase containing 100 mM Tris-HCl, 500 mM KCl (pH 8.3), 1.5 mM MgCl_2_, 200 µM of each deoxyribonucleoside triphosphate, and 0.025 U of Taq polymerase (Qiagen, USA). Amplification of DNA was performed using the Mastercycler personal PCR machine. For the Oliveira primers, the cycling conditions were as follows: initial denaturation for 5 min at 94°C; 35 cycles of denaturation for 30 sec at 94°C, annealing for 30 sec at 53°C, and extension for 1 min at 72°C; and a final extension for 5 min at 72°C. For the Zhang primers, the cycling conditions were as follows: initial denaturation at 94°C for 5 minutes; 10 cycles of 94°C for 45 seconds, 65°C for 45 seconds, and 72°C for 1.5 minutes; 25 cycles of 94°C for 45 seconds, 55°C for 45 seconds, and 72°C for 1.5 minutes; and a final extension at 72°C for 10 minutes. For amplification of the PVL gene, the cycling conditions were as follows: initial denaturation for 5 min at 94°C; 35 cycles of denaturation for 40 sec at 94°C, annealing for 40 sec at 53°C, and extension for 1 minutes at 72°C; and a final extension for 10 minutes at 72°C.

Data analysis was performed using Statistical Package for Social Sciences IBM SPSS 17.0 software (SPSS, Inc., Chicago, IL, USA). The significance of correlations for means was determined with the χ^2^ test and for independence with the Fisher exact test using GraphPad InStat 3.0 software (GraphPad Inc., San Diego, California, USA). Statistical significance was defined as a p-value <0.05.

## RESULTS

**Fig.1:**
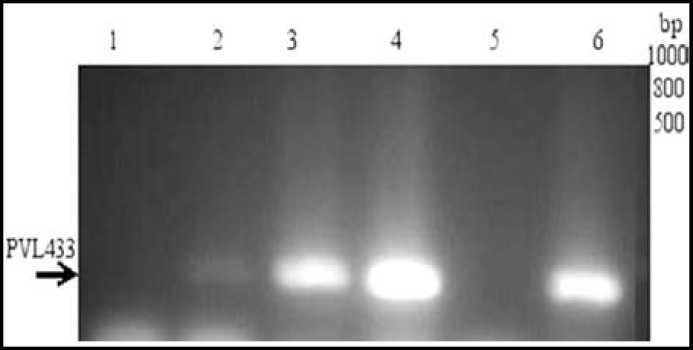
Multiplex PCR assay for SCC*mec* typing. Lane 1: negative control; Lane 2: positive control; Lanes 3, 4, and 6: 433-bp PVL gene fragment; Lane 5: isolate negative for the PVL gene; Lane M: 100-bp DNA ladder

**Fig.2:**
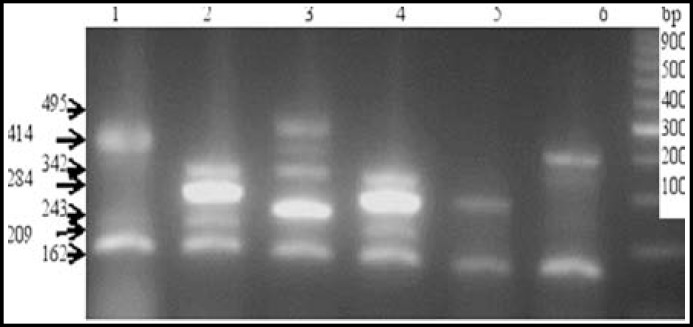
PCR detection of the PVL gene among selected MRSA isolates. Lane 1: negative control; Lane 2: positive control; Lanes 3, 4, and 6: 433-bp PVL gene fragment; Lane 5: isolate negative for the PVL gene; Lane M: 100-bp DNA ladder

Results from PCR analysis revealed that only 100 strains carried the* mecA *gene. The results from MRSA typing are shown in Table-II. Of the confirmed MRSA isolates, 3% were SCC*mec* type I; 9% were type II; 47% were type III, and 29% were type IV (Fig.1). Using Zhang primers, none of the isolates were identified as SCC*mec* type V. Twelve of the MRSA isolates could not be typed. Among the confirmed MRSA isolates, 19% harboured the PVL gene (Fig.2). PVL-positive isolates were mostly *SCCmec* type III (42.1%) and type IV (47.3%) (Table-II). There was a significant correlation between presence of PVL and SCC*mec* type IV (*P* = 0.0423). 

## DISCUSSION

In the past decade, the world has seen a steady increase in the incidence of MRSA. Methicillin resistance is conferred by the *mec*A gene, which is located on SCC*mec *and can be detected by PCR techniques for rapid and accurate identification of MRSA subcultures**.**^10^ In the present study, only 100 of 114 MRSA isolates were confirmed to carry the *mec*A gene. In combination with genotyping of the *S. aureus* chromosome, the SCC*mec* typing system has become an important technique for distinguishing between HA-MRSA and CA-MRSA. Most HA-MRSA infections are SCC*mec* types I, II, and III, while CA-MRSA infections are SCC*mec* types IV and V.^1^^,^^2^ In this study, two multiplex PCR systems were used: the first was developed by Oliveira and Lencastre**, **which identified four SCC*mec *types using characteristic genes in junkyard regions; the second was developed by Zhang *et al*.^3^^,^^4^ Both systems identified the *mec* and* ccr* region, although they differ slightly with respect to primer sequences and target genes.

**Table-I:** Primers used for amplification of SCCmec and PVL

	***Primers***	***Oligonucleotide sequence (5'–3')***	***Size (bp)***	***SCCmec type***
**Oliveira Primers**				
1	CIF2 F2	TTCGAGTTGCTGATGAAGAAGG	495	I
2	CIF2 R2	ATTTACCACAAGGACTACCAGC		
3	KDP F1	AATCATCTGCCATTGGTGATGC	484	II
4	KDP R1	CGAATGAAGTGAAAGAAAGTGG		
5	MECI P2	ATCAAGACTTGCATTCAGGC	209	II, III
6	MECI P3	GCGGTTTCAATTCACTTGTC		
7	DCS F2	CATCCTATGATAGCTTGGTC	342	I, II, IV
8	DCS R1	CTAAATCATAGCCATGACCG		
9	RIF4 F3	GTGATTGTTCGAGATATGTGG	243	III
10	RIF4 R9	CGCTTTATCTGTATCTATCGC		
11	RIF5 F10	TTCTTAAGTACACGCTGAATCG	414	III
12	RIF5 R13	GTCACAGTAATTCCATCAATGC		
17	MECA P4	TCCAGATTACAACTTCACCAGG	162	*mecA*
18	MECA P7	CCACTTCATATCTTGTAACG		
**Zhang Primers**				
19	Type II-F	CGTTGAAGATGATGAAGCG	398	SSmec II
20	Type II-R	CGAAATCAATGGTTAATGGACC		
21	Type III-F	CCATATTGTGTACGATGCG	280	SCC*mec *III
22	Type III-R	CCTTAGTTGTCGTAACAGATCG		
23	Type V-F	GAACATTGTTACTTAAATGAGCG	325	SCC*mec *V
24	Type V-R	TGAAAGTTGTACCCTTGACACC		
**PVL Primers**				
25	PVL-1	ATCATTAGGTAAAATGTCTGGACATGATCCA	433	PVL
26	PVL-2	GCATCAAGTGTATTGGATAGCAAAAGC		

**Table-II:** Frequency of *SCCmec* types and PVL among MRSA isolates

**MRSA clinical isolates**						
	***I***	***II***	***III***	***IV***	***V***	***Untypeable***
SCC*mec* (100)	3 (3%)	9 (9%)	47 (47%)	29 (29%)	-	12 (12%)
PVL (19)	0	0	8 (42.2%)	9 (47.3%)	-	2 (10.5%)

In the present study, among the 100 confirmed MRSA strains, 3% were SCC*mec* type I; 9% were SCC*mec* type II; 47% were SCC*mec* type III, and 29% were SCC*mec* type IV. We did not detect any SCC*mec* type V. The distribution of SCC*mec* types is reported to vary by region worldwide. For instance, SCC*mec* types I to III are the most frequent nosocomial MRSA strains in the world.^11^^,^^12^ In the present study, the majority of the hospital isolates were SCC*mec* type III. Similarly, SCC*mec* type III strains have been reported to be predominant in isolates from Saudi Arabia and other Asian countries**,** while SCC*mec *type II strains are predominant in Japan and Korea and are uncommon in Saudi Arabia, Philippines, Korea, and Japan.^13^

The incidence of CA-MRSA has increased and is a potential cause of healthcare-associated infections in Saudi Arabia**.**^14^ In a study conducted in the Makkah region, located on the West coast of Saudi Arabia, CA-MRSA accounted for 15.8% of all MRSA isolates.^14^

In the present study, we did not detect SCC*mec* type V isolates using Zhang primers. Similar results were reported by Budimir *et al*.^15^ Some authors have proposed using PCR with a single primer pair for the detection of type V, however, this method would require four separate reactions, which may not be suitable for large-scale analysis.^4^

In the present study, 12% of MRSA isolates could not be classified into any of the described SCC*mec *types. Similar data have been previously reported; Oliviera *et al*. reported that 8% of isolates were non-typeable for SCC*mec*.^3^

Some studies have reported the spread of CA-MRSA SCC*mec* type IV strains in hospital settings in Europe, United States, and Switzerland.^16^^,^^17^ CA-MRSA is most likely carried in the upper respiratory tract or various cutaneous and mucosal sites and introduced into the hospital by patients or nursing staff.^18^^,^^19^

Another genetic feature of CA-MRSA is the high prevalence of the PVL gene, which encodes a cytotoxin that causes leukocyte destruction and tissue necrosis. PVL-producing strains are associated with severe skin and soft-tissue infections and necrotizing pneumonia.^19^ In fact, the PVL gene is a stable marker of CA-MRSA and is largely absent from HA-MRSA strains**.**^6^ In the present study, 19% of isolates harboured PVL genes. Similarly, a study from the Netherlands reported that 15% of isolates were positive for PVL.^20^ Higher rates were reported in Algeria and while lower rates were reported in England.^21^^,^^22^

Recently, the genomic sequence of a CA-MRSA isolate indicated the presence of both SCC*mec* IV and PVL.^23^ Most CA-MRSA infections have been associated with strains bearing the SCC*mec* type IV element and PVL genes.^6^ Recently, a case series revealed that PVL-negative CA-MRSA may also cause severe necrotizing pneumonia, suggesting the presence of other virulence factors responsible for necrotizing pneumonia**.**^24^ In this study, we identified a statistically significant correlation between the presence of PVL and the SCC*mec* type IV gene (*P* value: <0.05). Moussa *et al*. studied the genotypes of MRSA from Riyadh and found that CA-MRSA strains harboured the SCC*mec* type IV element and PVL genes**.**^25^ Furthermore, Moroney *et al.* reported that 23 of 25 (92%) SCC*mec*-type-IV isolates were positive for PVL**.** Similar results were also obtained by Vandenesch *et al.,* who reported that the PVL gene and SCC*mec* IV were shared by all CA-MRSA isolates from three continents.^26^

Based on our findings, we conclude that over 50% of *S. aureus* isolates were MRSA in Makkah. Screening and isolation of MRSA-positive patients is essential in order to control the transmission of MRSA in both hospitals and the community. SCC*mec* types I–IV were identified among the *S. aureus* isolates, while type V was not detected in this study. There was a statistically significant correlation between the presence of the PVL gene and SCC*mec* type IV. The increasing incidence of virulent MRSA strains in both hospitals and the community highlights the importance of their rapid identification in order to appropriately control infection.
